# Survey datasets on categories of factors militating against safety practices on construction sites

**DOI:** 10.1016/j.dib.2018.06.101

**Published:** 2018-07-03

**Authors:** Kunle E. Ogundipe, Babatunde F. Ogunbayo, Adekunle M. Ajao, Uyoyoghene L. (Nee Agba) Ogundipe, Patience F. Tunji-Olayeni

**Affiliations:** a Department of Building Technology, Covenant University, Ota, Ogun State, Nigeria; b Department of Accounting, Covenant University, Ota, Ogun State, Nigeria

**Keywords:** Cause of accidents, Construction projects, Factors militating safety practices, Safety practices

## Abstract

The causes of occupational accidents have been classified into unsafe conditions and unsafe behaviour. Interestingly, numerous authors have contributed to the issues of safety practices in managing building production process with different views on factors causing construction accident and insensitiveness to safety practices, but there have been a little efforts to bring together major causes and factors militating against safety practices in unified manners. Therefore, all identified forty nine factors from literature review [1], [2], [3], [4], [5], [6], [7], [8], [9], [9], [11], [12], [13], [14], [15], [16], [17], [18], [19], [20], [21], [22], [23], [24], [25], [26], [27], [28], [29], [30], [31], [32] were brought together and grouped into five different categories. Descriptive statistics were performed on the data to rank these factors as affected workmen on construction sites. The results were presented in figures, text file and tables using Mean Score. The data presented in this study were enable construction managers to standardize project risks assessment and management.

**Specification Table**

**Table:** 

Subject area	Building and Civil Construction.
More specific subject area	Construction safety practices
Type of data	Tables, figures and text files
How data was acquired	66 copies of structured questionnaire were retrieved out of 75 survey data administered and simple statistical methods were used for the comprehensive analyses.
Data format	Raw data obtained from field survey
Experimental factors	Random sampling of different professionals working on construction sites in the study area. The data gotten were analyzed using SPSS and Microsoft Excel by ranking its Mean Score (MS) and they are presented in tables, figures and text files. Data gotten from the survey were measured on five-point Likert scale 5=Strongly Agreed, 4=Slightly Agreed, 3= Agreed, 2=Disagree, 1=Strongly Disagreed
Experimental features	Data were obtained through structured questionnaire to elicit needful information from different professionals working on Construction sites in the study area. Secondary data were gotten from extensive review of articles, conference papers, working papers and thesis that were relevant to this research topic.
Data source location	Lagos State, Nigeria.
Data accessibility	The research data are available within the article.

**Value of the data**

• The data pointed out different categories of factors militating against safety practices, the understanding of the data will enable government and policy makers in decision making and implementation in enhancing construction safety practices.
• This data will be helpful in any research that relates to construction safety practices in developing countries in order to establish measures for curbing factors militating against construction safety practices.
• The survey questionnaire will be useful in analyzing and averting anticipated project risks at planning stage and it will enable projects team to state the degree of confidence at which construction projects could be executed.
• The data will also serves as benchmark to compare findings of factors militating against construction safety practices from other developing countries.

## 1 Data

**Fig. 1:**
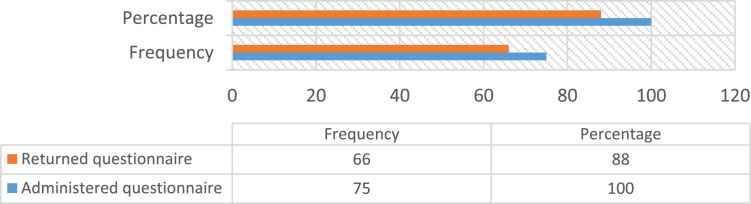
Survey response rate.

**Fig. 2:**
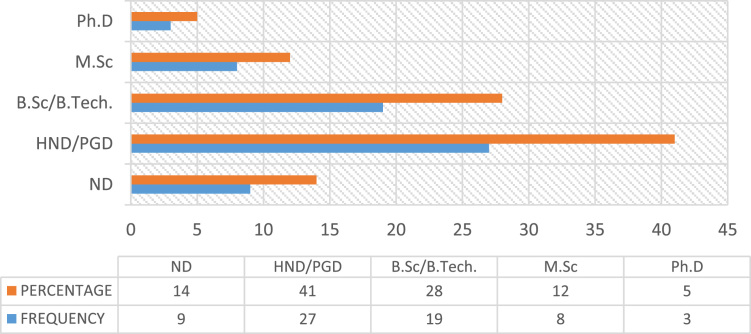
Education background of the respondents.

**Fig. 3:**
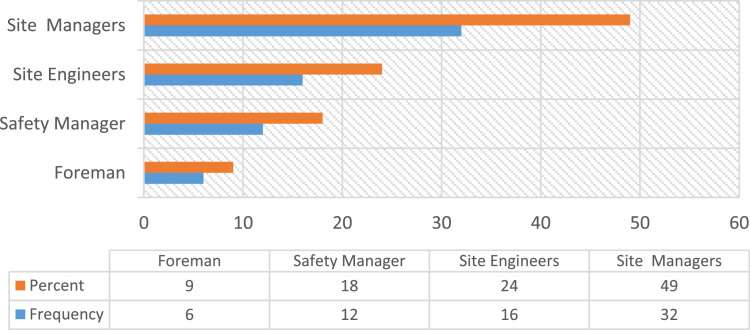
Category of operation of the respondents.

**Fig. 4:**
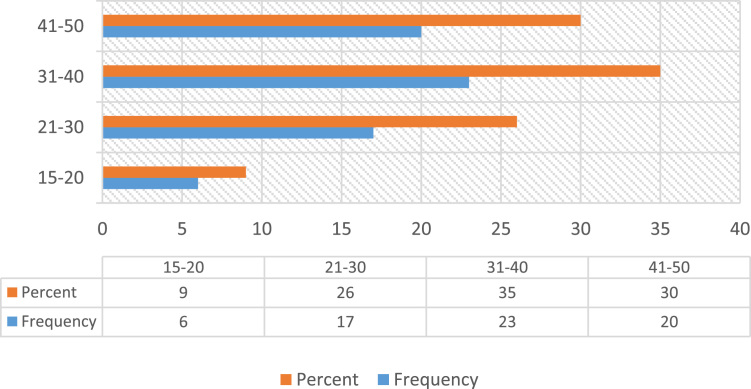
Age of the respondents.

**Fig. 5:**
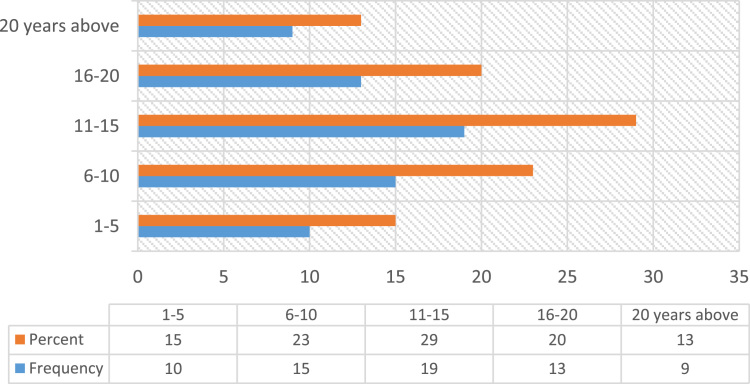
Respondents years of experience.

**Fig. 6:**
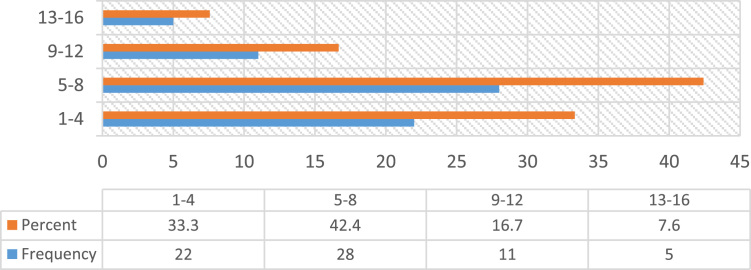
Professional׳s workmen ratio.

Construction accidents remained an ongoing concern in the developing countries, despite the level of awareness in promoting Occupational safety practices over the decades [23], [25]. Safety practice is anchored on workers behavior regarding safety provisions and conducts that guide workers attitude when carrying out their tasks at work in order to reduce or even eliminate accidental losses and injuries [33]. Prior to the presentation of this data article, adequate information and variables tested in the data were collated from the published and unpublished previous studies on issues regarding safety practices, safety performance and productivity, challenges facing the implementation of health and safety practices, compliance and management of safety on the construction sites [1], [2], [3], [7], [9], [13], [14], [16], [18], [23], [25], [27], [30] as they were considered relevant to the subject of construction health and safety practices. The data of this article were gotten from selected construction sites in Lagos State, Nigeria. Fig. 1 Showed survey response rate, as 66 copies of questionnaire were retrieved and analyzed justifying 88% response rate. Fig. 2 showed the education background of the respondents with the clear indication that respondents of this data article have required education background needed for this study. Fig. 3 revealed category of operation of the respondents, largest percentage of the respondents’ were site managers, some of them perform dual functions as a safety manager on their sites. Fig. 4 indicated the age of respondent׳s ranges between 15–50 years old. Fig. 5 showed respondent׳s years of experience. Both the age and years of experience of the respondents were relevant to this study in order to get accurate responses for the variables tested. Fig. 6 explained workmen ratio on sites, this is also believed to be one of the neglected factor responsible for low compliance to safety practices when site managers have more than enough workmen to supervise during working period.

## 2 Experimental design, materials and methods

**Fig. 7:**
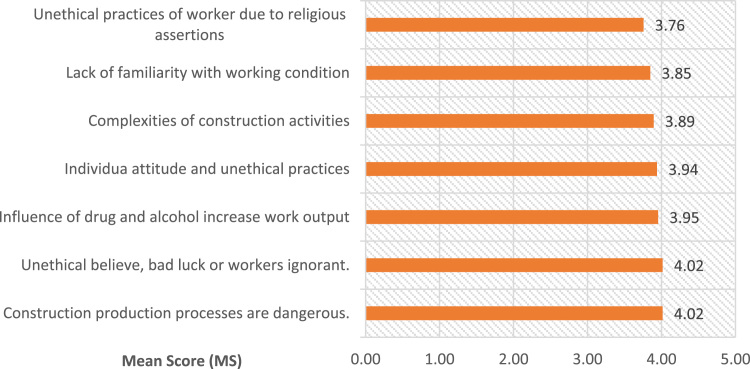
Workmen made belief factors militating safety practices.

**Fig. 8:**
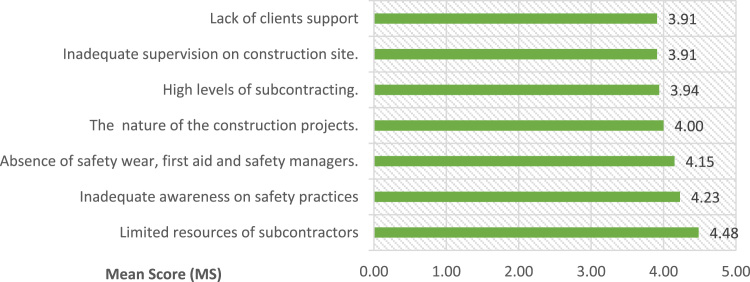
Management structure factors militating safety practices.

**Fig. 9:**
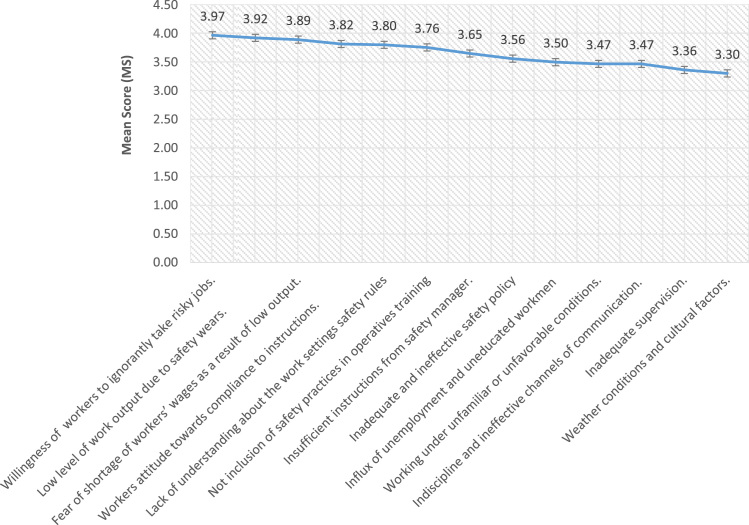
Factors related to work environment.

The data for this study covered medium and large scale construction firms operating in Lagos State. Lagos State remained one of fastest growing state in Africa, it is also a coastal zone with a tremendous increase in modern construction activities and development such as: Eko Atlantic city, Lekki free trade zone (Dangote petroleum refinery and Lekki deep sea port) and Lagos Island international airport [22], [23]. The data ranked categories of factors militating against safety practices on construction sites thereby causing accidents as collated and established from the extensive literature review. The identified forty nine factors militating against safety practices in developing nations as evidence in Lagos State, Nigeria were grouped into five namely i. workmen made believed factors, ii. Management structure factors, iii. Operative’ shortage of technical skills, iv. Factors related to safety law enforcement, and v. factors related to work environment. From category one as presented in Fig. 7**,** seven variables were identified from [10], [12], [14], [26]. Fig. 8**,** highlighted seven variable from [18], [23], [25], [26], [27], [28], [32] under second category. Third category of factors militating against safety practices were presented in Table 1. [4], [8], [9], [19], [22], [24], [29], [31]. Table 2 presented thirteen factors related to safety law enforcement under fourth category [2], [3], [8], [9], [17], [20]. The fifth category were presented in Fig. 9 [3], [5], [6], [13], [15], [16], [17], [21], [30].

**Table 1:** Operatives shortage of technical skills as factors militating against safety practices.

Variables	Mean Score	Ranking
Problem of adaptability of workers to safety practices as it was against their traditional practices	3.89	1st
Inadequate of required experience of the Safety manager to manage workmen	3.88	2nd
Wide gaps of workmen ratio between the supervisor and artisans	3.86	3rd
Limited technical and financial resources to identify and control risks and operational hazards.	3.83	4th
Lack of safety education and commitment from construction professionals	3.73	5th
The use of migrant workers on construction sites.	3.73	6th
Lack of training on key issues pertaining health and safety consciousness	3.73	6th
Manual handling of heavy materials and component	3.55	8th
Lack of proper documentation of accidents on site	3.42	9th

**Table 2:** Factors related to safety law enforcement.

**Variables**	Mean Score	Ranking
Corruption due to improper enforcement of laws and regulations.	4.14	1st
Low enforcement of construction labour safety law	4.12	2nd
Absence of safety monitoring system on construction sites	3.97	3rd
Inadequate safety by-laws and standards	3.89	4th
Absence of company׳s safety regulations and policies.	3.83	5th
Epileptic enforcement mechanism	3.77	6th
Wide ratio between safety manager and workmen	3.64	7th
Weak safety regulatory authority or non-existent	3.50	8th
Weak statutory OSH regulations and provisions.	3.48	9th
Limited legislation governing Health and Safety practices	3.47	10th
Lack of attention for general conditions of workers	3.32	11th
Inadequate support from professionals body for enforcement	3.30	12th
Frequent omission of workmen from insurance policy	2.91	13th

Since there are no accurate records on number of construction activities in the study area, the study adopted random sampling techniques in selecting population for the study. 75 copies of structured questionnaire were circulated to construction professionals with vast knowledge and proven years of experience to survey their opinion. The study got 88% response rate which are 66 copies from the total copies of questionnaire administered and they were fully analyzed. The survey data were measured on five-point Likert scale Strongly Agree =5, Slightly Agreed =4 Agreed =3, Disagree =2 Strongly Disagreed =1. The identified forty nine variables were designed into closed ended questionnaire, ranked with Mean Scores and presented in figures and tables using Microsoft Excel to allow easy replication of this data.

The ranking of this factors have categorized the forty nine factors militating against safety practices as evidence in Lagos State, Nigeria. Details of the previous studies as related to this data article could be found in [1], [2], [3], [4], [5], [6], [7], [8], [9], [10], [11], [12], [13], [14], [15], [16], [17], [18], [19], [20], [21], [22], [23], [24], [25], [26], [27], [28], [29], [30], [31], [32], [33]**,** while the research method adopted is similar to that of [34].
